# A Case of Isolated Duodenal Varices Secondary to Chronic Pancreatitis with Review of Literature

**DOI:** 10.4021/gr249w

**Published:** 2010-11-20

**Authors:** Venugopala Bommana, Prasun Shah, Michael Kometa, Rawan Narwal, Prashant Sharma

**Affiliations:** a St. Vincent Mercy Medical Center, Toledo, Ohio, 2213 Cherry Street, Toledo, Ohio 43608, USA; b St. Vincent Charity Hospital, Cleveland, Ohio, 2351 East 22nd Street, Cleveland, OH 44115, USA

**Keywords:** Isolated duodenal varices, Hemorrhagic complications of pancreatitis, Hemetemesis, Superior mesenteric vein obstruction

## Abstract

An unusual case of upper gastrointestinal hemorrhage due to an isolated varix involving the 2nd part of the duodenum is presented here. The varix was the result of Chronic Pancreatitis induced the superior mesenteric vein obstruction. The diagnosis was made preoperatively by upper gastrointestinal endoscopy and selective mesenteric angiogram. Patient was treated successfully with Mesocaval shunt surgery between the superior mesenteric vein and the inferior vena cava using a 10 mm Dacron graft. This is the unique case showing hemorrhagic complication of Chronic Pancreatitis due to the superior mesenteric vein obstruction.

## Introduction

Chronic pancreatitis is a major health hazard and since long we know different complications due to it. Here we have presented a case of isolated duodenal varices which we found incidentally in our patient who has presented with hemetemesis and at the end of long investigational battery we found that patient has superior mesenteric obstruction secondary to adhesions in the peripancreatic region. In literature there are very few cases reported showing bleeding duodenal varices, most of them are in the duodenal bulb which are part of portal hypertension. In our patient there was no evidence found for portal hypertension and duodenal varices were in the second portion of the duodenum. Patient had superior mesenteric obstruction secondary to chronic pancreatitis which is the unique presentation of the complication.

## Case Report

A 33 years old Caucasian male presented to the emergency room of St.Vincent Mercy Medical Center at Toledo, Ohio with a history of intermittent hemetemesis for last couple of weeks which has increased in severity during the last 24 hours, progressing to 4 episodes of a cup full of blood. He also had complaints of light headedness and dizziness. He had no history of hemetochezia, melena, fever, chills, constipation, diarrhea, abdominal pain or nausea. He had no recent NSAIDs use, headache, chest pain, shortness of breath or palpitation.

Patient had past history of Diabetes Mellitus type 1 and is on Insulin. Patient had history significant for Gallstone pancreatitis which resulted in Pseudocyst formation. Patient had undergone Cholecystectomy and Cystojejunostomy in 2007. He had history of Spinal Stenosis, right knee surgery and Tonsillectomy. Patient was allergic to Avandia, Fentanyl, and Metfromin. Patient was on Tramadol, Neurontin, Insulin, Zolpidem and Omeprazole. Patient had no history of Gastrointestinal (GI) disease in family. His father had diabetes and mother had COPD and pernicious anemia. Patient was a non smoker, non alcoholic and denied using any illicit drugs. His bowel and bladder functions were unaltered.

On presentation his vitals were Temp: 36.3 °C, Pulse: 114/min, Blood pressure: 102/54 mm of Hg, Oxygen saturation: 99% on room air and Respiratory rate: 16/min. On physical examination he was conscious, coherent, pale looking and weak with warm and moist skin without dermatosis. No signs of Jaundice or generalized Lymphadenopathy were found. He had no obvious spinal or limb deformities. He had normal pulses in all extremities without any signs of cyanosis. His oral cavity was normal without ulcerations. His chest was symmetrically expanding with good breath sounds and without labored breathing. He had adynamic precordium with apex beat located in 5^th^ intercostal space at midclavicular line. He had normal heart sounds without any murmur. His abdomen was soft and flat. He had tenderness in epigastric region. No organomegaly or masses were found and bowel sounds were normal. Patient had normal neurological examination with grossly intact cranial nerves and normal reflexes. Pupils were equal and reacting to light normally.

**Figure 1:**
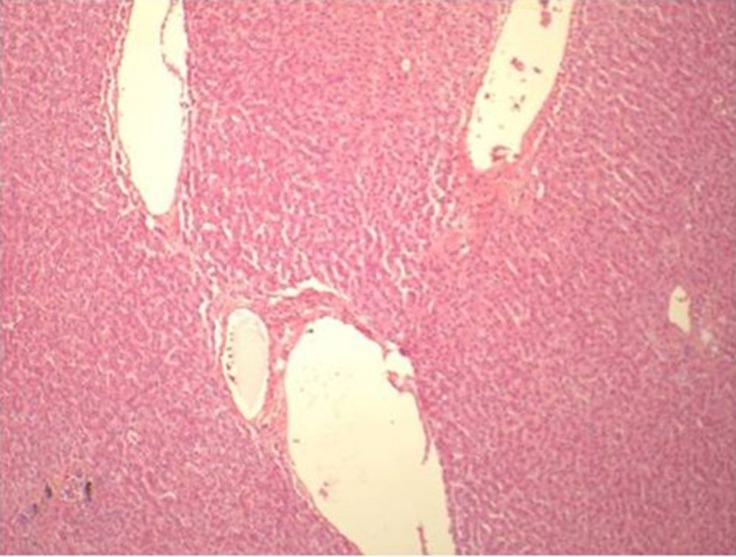
Liver Biopsy showing normal architecture

His initial investigations were Hemoglobin 6.3 gm/dl, Hematocrit of 18.7%, MCV: 88.8 fL, White cell count 8.4 k/ul (Neutrophils 84, Lymphocytes 10, Monocytes 6, Eosinophils 0, Basophils 0), Platelet count: 327 k/ul, Sodium: 137 meq/l, Potassium: 4.7 mEq/l, Bicarbonate: 24.5 mEq/l, Chloride: 103 mEq/l, Blood Urea Nitrogen: 35 mg/dl, Creatinine: 0.59 mg/dl, Glucose: 231 mg/dl, Bilirubin: Direct: 0.12 and total 0.28 mg/dl, Proteins total: 4.4 g/dl, Albumin: 2.7 g/dl, AST: 31 units/L, ALT: 44 units/l, Calcium: 7.9 mEq/l, Alkaline phosphatase: 53 units/l, Lipase: 23 units/l, pCO_2_ 46, pO_2_ 84, pH 7.34, PTT: 23.4 (20.3-30.9 sec), Prothrombin time: 12.5 (9.7-12 sec), INR: 1.2, Fibrinogen: 420(140-420 mg/dl). Hepatitis serology and ANA screen were negative. He had undergone Liver biopsy was performed during surgery which was found normal without any changes of cirrhosis of liver (Fig. 1).

**Figure 2:**
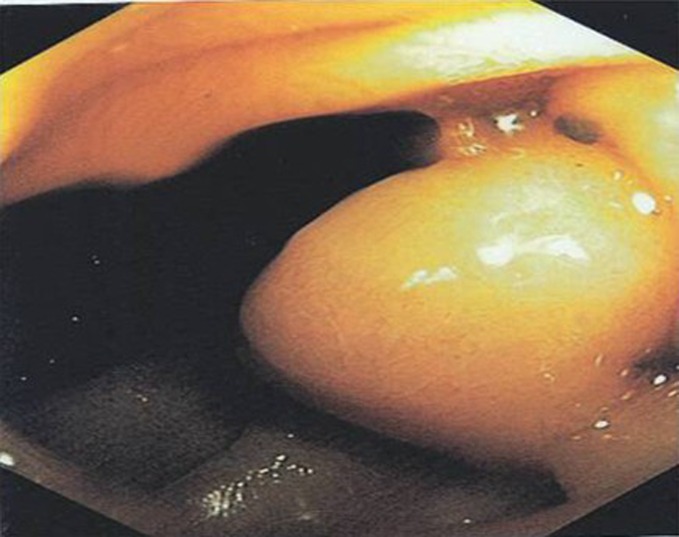
Duodenal varix in EGD

On upper GI endoscopy (EGD) esophageal and stomach mucosa were found normal without any active sites of bleeding. Fundus was clearly seen without varices. Pylorus was patent and the duodenal bulb was normal. The major papilla was identified in the descending duodenum and had normal appearance. Just distal to the major papilla in the 2^nd^ portion of duodenum, there was a large submucosal bulge suspicious for an ectopic duodenal varix (Fig. 2). On withdrawal of the scope findings were confirmed.

Abdominal and pelvic CT scan with contrast showed massive gastric distention. Gastric outlet obstruction was suspected. Pancreatic Pseudocyst in the tail of the pancreas had significantly increased in size from the previous study. Changes of chronic pancreatitis were present.

**Figure 3:**
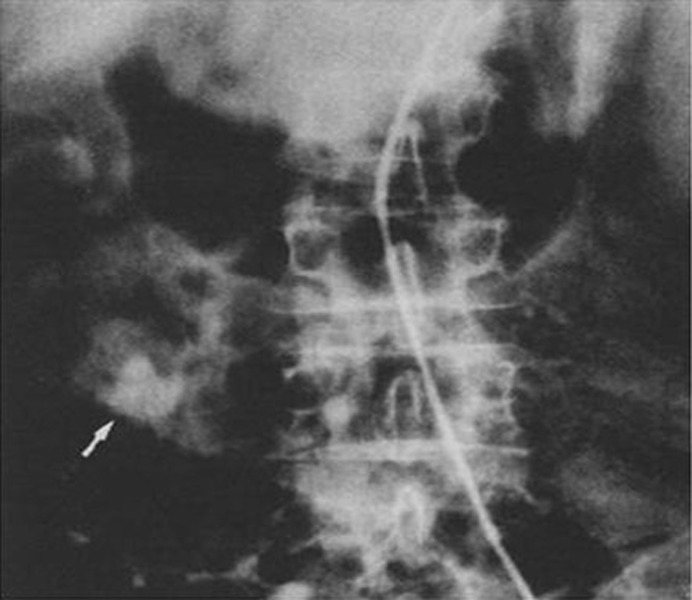
Venous phase of the superior mesenteric artery injection showing varices in the region of the duodenum

Mesenteric Arteriogram showed slow contrast filling of the superior mesenteric vein with significant limiting of flow. Superior Mesenteric Vein obstruction was suspected. Multiple venous varicosities around the distal stomach and the pancreatic head region were found (Fig. 3). Possibility of at least partial thrombosis of the Superior Mesenteric Vein (SMV) near the pancreatic head region was suspected secondary to pancreatitis. Splenic vein was found of normal caliber without any varicosities.

Patient had undergone end to side Mesocaval Anastomosis with a Dacron 10 mm graft between the Superior Mesenteric Vein and the Inferior Vena Cava along with adhesionolysis and liver biopsy. During surgery no varicosities found in the gastric and esophageal region or no dilation found in the splenic or inferior mesenteric venous system. There were multiple adhesions found in the tail region of pancreas possibly because of previous Cystojejunostomy in that area. Similarly many adhesions found in the peripancreatic region most likely due to changes of chronic pancreatitis. Patient was discharged without any complications and found asymptomatic in the follow up visits with normal hemoglobin level and without any evidence of bleeding. Considering the young age of the patient and relatively absent varices at other sites Mesocaval shunt surgery was chosen as a treatment over Transjugular Intrahepatic Portosystemic Shunt (TIPS) and duodenal resection.

## Discussion

Duodenal varices were initially recognized as a source of gastrointestinal hemorrhage by Alberti who described the radiologic findings in three cases in 1931 [1, 2]. Since then approximately 105 cases has been noted in the literature. Out of them in our knowledge one case is been reported for the pancreatitis induced small bowel varices by Brian et al in 1992 [3]. Most of the cases reported for isolated duodenal varices were referable to Portal Hypertension, approximately 30% of them are resulting from cirrhosis of liver [4-6]. These percentages vary geographically as evidenced by Al-Mofarreh’s series of 13 patients with Schistosomiasis [7]. Other important and rare etiologies of Duodenal varices are mentioned in Table 1.

**Table 1:** Etiology of Duodenal varices in world Literatures

Cirrhosis of Liver [1, 4-6]
Thrombotic disorders and Coagulopathies [4]
Schistosomiasis [7]
Pancreatitis and Cholangitis [3, 8]
Omphalophlebitis (surgical catheterization of Umbilicus in adults) [8-10]
Pancreatic and periportal tumors [8]
Mesenteric Metastasis [4, 11]
Surgical Procedure or Trauma [4, 11]
Visceral Hemangiomata (Klippel-Trenaunay-Weber syndrome) [12-16]
Vascular Anomalies (Occlusion of SMV by abnormal Ileocecal Artery) [12-16]
Retroperitoneal Fibrosis [8]

Duodenal varices have a high bleeding rate and a recent review of 16 reported Cases showed that 12 patients (75%) had massive hemorrhage (mean 10.4 + 3.8 units of blood) and required emergency surgery. Interestingly, 75% of patients in this review had previous episodes of gastrointestinal hemorrhage, thus raising the possibility that duodenal varices have a tendency to rebleed [17-19].

Duodenal varices are considered dilated veins of Retzius around the pancreaticoduodenal region [20, 21]. Duodenal varices usually consist of a single vessel with associated afferent and efferent vessels which form a retroperitoneal portosystemic shunt [21, 22]. The afferent vessel arises either from the superior or inferior pancreaticoduodenal vein or from the SMV [21, 22]. The efferent vessel is thought to arise from one of the many retroperitoneal veins and commonly drains into the inferior vena cava. The varices usually lie in the posterior wall of the duodenal bulb [20, 21]. Distribution of varices in different areas of the duodenum and its association with varices in other regions is presented in Table 2. According to available data most common site of varices in duodenum is duodenal bulb followed by second part of the duodenum.

**Table 2:** Review of Cases of Duodenal Varices [23]

Part of Duodenum	Duodenal Bulb	48
	2nd portion	12
	3rd portion	3
	4th portion	1
	DuodenoJejunal Junction	3
	Not Specified	38
		
Associated Varices	Esophagus	25
	Esophagus and Stomach	2
	Esophagus, Stomach and Jejunum	1
	Not recognized	5
	Not Specified	72

We hypothesize that the bouts of pancreatitis led to progressive fibrosis of the peri pancreatic tissues and eventual occlusion of the Splenic and Superior Mesenteric veins [3]. Esophagogastric varices usually result from Splenic vein thrombosis and development of portosystemic collaterals in the short gastric veins. This is commonly called “left-sided portal hypertension” or “Sinistral Hypertension” [24-26]. Occlusion of the portal vein from pancreatitis can then lead to generalized portal hypertension and subsequent esophageal or gastric variceal bleeding [3, 24, 25]. Thrombosis of the superior mesenteric vein may lead to elevated splanchnic venous pressure and the potential for variceal bleeding, although this complication has not been reported previously [3, 24, 25, 27-29]. In patients with pancreatitis, SMV occlusion may result from the progression of the fibrosis to the peri pancreatic tissues [3, 24, 25, 27-29]. Most patients, however, do not have bleeding complications, and the diagnosis of mesenteric vein occlusion is usually made during exploratory laparotomy for other reasons or on pre-operative angiography for other complications of pancreatitis [3, 24, 25, 27-29].

Diagnosis of duodenal varices is almost always made during endoscopy for investigation of GI bleeding [4]. The diagnosis has also been made on barium studies, spleenoportography, angiography and laparotomy [4, 30]. It is not unusual to use more than one of these modalities to confirm the diagnosis. When duodenal varices are diagnosed by endoscopy, 60% have varices elsewhere in the GI tract and 50% of these are gastoresophageal. 40% of patients with portal hypertension have duodenal varices at angiography. But they are clinically not so significant because they rarely penetrate the submucosa [30-32]. CT scan with contrast and MRI are not routinely used for the diagnosis of ectopic varices [29]. Ultrasound and color duplex has some informative and diagnostic values when blood flow is more than 5cm/second. Though its efficacy in diagnosis of intra-abdominal ectopic varices is not known and usefulness in investigation of Upper GI bleeding is to be evaluated [33, 34]. Some reports mention its use in diagnosis of patency of shunt following shunt surgeries.

Capsule endoscopy (CE) has revolutionized examination of the small bowel. Currently, the most common indication for CE is GI bleeding of obscure etiology [12]. For this indication, the yield of CE is reportedly about 67% [12, 35]. The most common findings include angioectasia, fresh blood, ulceration, tumor and varices. The mucosal color of small bowel varices may differ minimally from that of the surrounding mucosa [12, 35-37]. Technetium scintigraphy is been used in some cases for diagnosis of GI bleeding and needs further evaluation. Selective mesenteric angiography and spleenoportography have sensitivity of 50% [19, 32]. Angiographic and spleenoportographic examination are helpful though, in that it will clearly define the anatomy, which may include duodenal and esophageal components, and proves useful in planning the operative approach [19, 32].

Treatment of small bowel variceal bleeding is directed toward relieving the segmental elevated pressures and is based upon the etiology of the varices [3]. Treatments of bleeding from ectopic varices include local treatment performed endoscopically, radiologically, surgically with portal decompressive surgery or TIPS [38]. The efficacy of pharmacological treatment with beta blockers has never been evaluated [38]. Endoscopic sclerotherapy has been tried for duodenal, anorectal and gastric varices but almost invariably rebleeding occurred and ulcers following sclerotherapy may result in life threatening hemorrhage [39, 40]. Variceal embolization after portal vein catheterization can control bleeding but rebleeding is not unusual due to the development of new collaterals feeding the ectopic varices [38, 41]. Surgical ligation of varices is also a temporary measure that does not allow definitive control of variceal bleeding; this is not surprising as none of these methods relieves the portal hypertension [42]. Balloon Temponade in case of varices in the second portion of duodenum is out of question.

TIPS is a hemodynamic equivalent of a side to side small diameter Portocaval shunt, it is much less invasive than surgery and can be performed even in patients with decompensated liver disease [38, 43-45]. TIPS has been widely used for the treatment of bleeding from gastoresophageal varices as well as refractory ascites. This procedure is safe with a 1-2% incidence of life threatening complications. The rate of hepatic encephalopathy is much lower than Portocaval shunt surgery. Shunt dysfunction due to progressive stenosis following pseudointimal hyperplasia inside the stent is a significant problem. Accordingly shunt function must be followed closely with duplex Doppler ultrasound [43-49]. Shunt catheterization and angiographic interventions are frequently needed. The use of stents covered with PTFE has been reported to markedly reduce the incidence of shunt stenosis and will probably represent a major advance in the future [41].

Portocaval shunt surgery has been used in some patients however the postoperative morbidity and mortality rates are high particularly in patients with decompensated liver disease [50]. Decompressive surgery is very efficient at stopping the rebleeding but it may precipitate liver failure or induce recurrent hepatic encephalopathy [34, 43]. In patients with bleeding secondary to portal hypertension treatment consists of portosystemic shunting and resection of the variceal segment [34, 50].

Our patient was treated successfully with Mesocaval shunt surgery [50]. In our case patient was young and had elevated pressure in the Splanchnic Circulation secondary to obstruction of the Superior Mesenteric vein. And as only superior mesenteric vein was narrowed due to chronic pancreatitis without other evidences of portal hypertension TIPS was not considered. We performed shunt surgery instead of resection of the 2^nd^ part of the duodenum as we believed that resecting the affected portion was difficult due to presence of adhesions and did not offer complete solution in this case. In comparison we found that relieving the segmental pressure was a better option.
